# Predictors for Upper-Limb Functional Recovery Trajectory in Individuals Receiving Stroke Rehabilitation: A Secondary Analysis of Data from Randomized Controlled Trials

**DOI:** 10.3390/ijerph192416514

**Published:** 2022-12-08

**Authors:** Batsaikhan Buyandelger, Yu-Wen Chen, Yi-Chun Li, Chia-Jung Lin, Chia-Ling Chen, Keh-Chung Lin

**Affiliations:** 1 School of Occupational Therapy, National Taiwan University College of Medicine, 17, F4, Xu-Zhou Road, Taipei 100, Taiwan; 2 Department of Physical Medicine and Rehabilitation, Chang Gung Memorial Hospital at Linkou, 5 Fusing Street, Gueishan District, Taoyuan 333, Taiwan; 3 Graduate Institute of Early Intervention, College of Medicine, Chang Gung University, 259 Wenhua 1st Road, Gueishan District, Taoyuan 333, Taiwan; 4 Division of Occupational Therapy, Department of Physical Medicine and Rehabilitation, National Taiwan University Hospital, 7 Chung-Shan South Road, Taipei 100, Taiwan

**Keywords:** stroke, rehabilitation, prediction, recovery

## Abstract

Background: The objective of the study was to determine predictors for upper-limb functional recovery trajectory after occupational therapy in a population with chronic stroke. Methods: In this retrospective secondary analysis, Fugl–Meyer Assessment-Upper Extremity (FMA-UE) scores before and after intervention and at the 3-month follow-up were used to divide 105 participants with chronic stroke into three groups of recovery trajectories: fast (participants who reached an improvement of 7 after intervention), extended (those who reached an improvement of 7 at follow-up), and limited (those who did not reach an improvement of 7) recovery. Comparisons among the three groups were made in demographics, stroke characteristics, and baseline assessment scores. Logistic regression analyses were performed to determine predictors for group membership. Results: Time after onset of stroke and the baseline scores of FMA-UE, Stroke Impact Scale-Hand (SIS-Hand), Wolf Motor Function Test (WMFT)-Quality, WMFT-Time scores, Motor Activity Log-Amount of Use (MAL-AOU), and Motor Activity Log-Quality of Movement (MAL-QOM) scores were significantly different among the three groups. Univariate logistic regressions confirmed that SIS-Hand, WMFT-Quality, WMFT-Time, MAL-AOU, and MAL-QOM were significant predictors for both the fast versus limited recovery group membership and the extended versus limited group membership. Time after stroke onset and baseline FMA-UE were additional predictors for the fast versus limited recovery group membership. Conclusion: These findings may assist healthcare professionals in making optimal therapeutic decisions and in informing clients and caregivers about the outcomes of stroke recovery.

## 1. Introduction

Stroke is a leading cause of death and disability worldwide, and the economic costs of treatment and post-stroke care are substantial. The global estimated disability-adjusted life-years due to stroke exceeds 116 million [1]. Multidisciplinary team-based rehabilitation and supported discharge with home-based rehabilitation are effective interventions for reducing the odds of death and functional dependency after stroke [2,3,4]. However, the response to the rehabilitation of individuals with chronic stroke varies greatly from person to person [5]. Personal factors and clinical predictors of these heterogeneous recovery patterns over time are still being investigated.

According to the proportional recovery rule, most stroke patients achieve 70% of available improvement in the Fugl–Meyer Assessment-Upper Extremity (FMA-UE) subscale from the baseline score within 3 months [6]. Prediction studies to the proportional recovery rule suggested that the baseline score of FMA-UE was a predictor [7,8,9]. In addition to prediction studies of the recovery rule, predictors of minimal clinically important difference (MCID) changes in the FMA-UE were determined in acute [7,9,10], subacute [11], and chronic [5,11,12,13,14] phases. The baseline FMA-UE score and modified Rankin Scale were reported as predictors of an improvement in FMA-UE in acute stroke [10]. The hand movement scale and time since onset were reported as predictors in subacute stroke [11]. The hand movement scale, time since onset, tactile sensory, hand function, baseline functional independence measure (FIM), and baseline FMA-UE score were reported as predictors in chronic stroke [5,11,12,14].

However, the limited accuracy of current prediction models in predicting stroke outcome suggests more work is required to improve accuracy [15]. Recently, researchers defined novel recovery trajectory groups (fast, extended, and limited recovery) and investigated the association between baseline characteristics and recovery trajectory groups in a population with acute stroke [15]. The longitudinal cohort study demonstrated that baseline scores of the FMA-UE, National Institutes of Health Stroke Scale (NIHSS), Barthel Index, and total hours of physical and occupational therapy were significantly associated with trajectory group membership in their group of 40 participants with subacute stroke. The objective of this study was to apply the methods of predefined recovery trajectory in individuals with chronic stroke and receiving occupational therapy to investigate predictors for these recovery trajectories after occupational therapy.

## 2. Materials and Methods

This study was a retrospective secondary analysis of data collected for studies conducted by our research laboratory. The original clinical trials were registered on https://clinicaltrials.gov (NCT03773653 and NCT04978311; accessed on 1 October 2022). Data analyzed in this secondary analysis were from both projects. Available results and one of the protocols were published [16,17]. The research designs and methods of the original studies were approved by the Institutional Review Boards of all involved clinical settings. All participants provided informed consent at the time of participating in the original studies.

### 2.1. Participants

Participants in this secondary analysis were individuals with hemiplegia after stroke who were recruited to participate in the original studies from participating hospitals in Taiwan between December 2018 and March 2022. The inclusion criteria for the original studies were (1) at least 3 months after the onset of a first-ever unilateral cerebral stroke and (2) a baseline FMA-UE score >10. The exclusion criteria were (1) Modified Ashworth Scale score >3, (2) The Montreal Cognitive Assessment score <15, and (3) other neurologic or orthopedic disorders.

In the original studies, sample sizes were estimated based on the results of previous studies. Participants were recruited and randomized into one of the treatment groups using computer-generated random-sequence tables by research assistants. The participants received 18 sessions of occupational therapy for 90 min per day, 3 sessions per week, for 6 weeks in the hospital outpatient clinics. They received robot-assisted therapy, mirror therapy, or conventional rehabilitation. The interventions were delivered by well-trained and certified occupational therapists.

### 2.2. Outcome Measures and Potential Predictors

Trained raters (also certified occupational therapists) conducted outcome assessments before and after the intervention and at the follow-up 3 months after the intervention ended. During the study, the raters were blinded from the treatment group assignments and not allowed to discuss participants’ treatment allocation or procedures in any form with therapists and participants, and vice versa.

The observed upper-limb (UL) motor function, as assessed with the reliable and valid FMA-UE [18] was selected to define participants’ recovery trajectory. Similar to Kline et al. [15], we separated participants into three recovery groups. In their study, the fast recovery group demonstrated achievement of the FMA-UE MCID score after the 6-week intervention, the extended recovery group achieved the MCID at the follow-up measurement, and the limited recovery group achieved less than an FMA-UE MCID score at all measurement assessments. We selected a seven-point MCID for the FMA-UE because participants of this study were patients with chronic stroke [19].

To identify predictors of UL recovery trajectory in our participants, we compared demographics, stroke-related characteristics, and baseline UL assessment scores among the predefined recovery groups. The demographics collected were age, sex, and years of education. Stroke-related characteristics were side of hemiplegia, stroke diagnosis (hemorrhage or ischemia), and time after stroke onset. All participants of this study were right-handed before onset of stroke; therefore, the variable of side-of-hand dominance was excluded from the analysis. The baseline assessment scores included the NIHSS, FMA-UE [18], Motor Assessment Scale (MAS) [20], The Wolf Motor Function Test (WMFT) [21], Stroke Impact Scale Hand (SIS-Hand) subtest [22], Motor Activity Log (MAL)-Amount of Use (AOU) and Quality of Movement (QOM) subscales [23], and the Nottingham Extended Activities of Daily Living (NEADL) [24].

### 2.3. Statistical Analysis

Normality for all variables was checked with the Shapiro–Wilk test. Mean ± standard deviation values are presented for normally distributed variables and median (first quartile-third quartile) values are presented for non-normally distributed variables. Group differences in continuous variables were assessed using one-way analysis of variance or Kruskal–Wallis analysis. Categorical analyses are presented as number (%), and group differences were calculated using χ^2^ tests. Post hoc analysis of paired comparisons was conducted using Bonferroni tests or Wilcoxon rank sum tests. Univariate logistic regression analyses were then conducted to identify predictors for group membership. The significance (α) level was set at 0.05. The analysis was conducted by using IBM SPSS Statistics (RRID: SCR_019096) for Windows 26.0 software (IBM Corp, Armonk, NY, USA).

## 3. Results

### 3.1. Participant Characteristics

**Figure 1:**
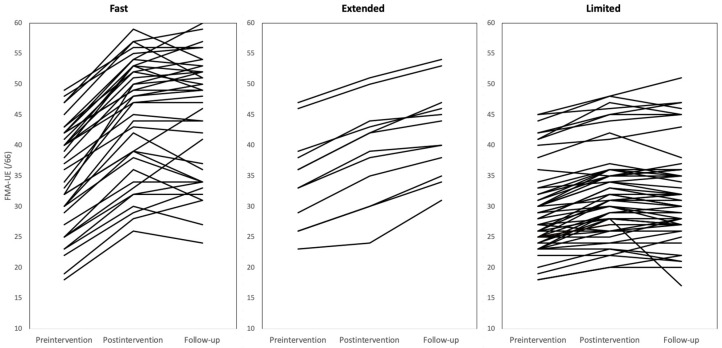
Line plots showing recovery trajectories by group. The x-axis shows time and the y-axis shows Fugl–Meyer Assessment-Upper Extremity subscale (FMA-UE) scores. The left plot shows the fast recovery group (n = 38), the center plot shows the extended recovery group (n = 12), and the right plot shows the limited recovery group (n = 55).

Of the 117 participants in the database, 12 were excluded due to missed FMA-UE follow-up measurement (n = 11) or mild initial arm impairment with a baseline FMA-UE score of >50 (n = 1). The 105 participants who met the study criteria were divided into three recovery groups: fast recovery (n = 38 [36.19%]), extended recovery (n = 12 [11.43%]), and limited recovery (n = 55 [52.38%]) (see Figure 1). Mean age was 55.67 ± 11.42 years, male/female sex ratio was 70/35, median year of participant education was 12 (9–14) years, percentage of right-side hemiplegia was 58.10% (61 of 105), percentage of hemorrhagic stroke was 44.76% (47 of 105), and median time between stroke onset and study participation was 14 (7–31) months (Table 1).

### 3.2. Comparisons of Baseline Measurement Scores among Recovery Groups

Table 2 shows the results of group comparisons. Demographic variables were not significantly different between the predefined recovery groups. Time after stroke was significantly different across the three groups (H(2) = 10.48, *p* = 0.005) and was shorter in the fast recovery group and the extended recovery groups. Pairwise tests of the mean rank differences between groups showed that time after stroke onset was significantly shorter in the fast recovery group compared to the limited recovery group (*p* = 0.002), and similar between the extended recovery group and the limited recovery group (*p* = 0.072).

The Kruskal–Wallis test showed group differences in baseline characteristics, including FMA-UE (*p* = 0.007), WMFT-Quality (*p* < 0.001), WMFT-Time (*p* = 0.002), SIS-Hand (*p* = 0.007), MAL-AOU (*p* = 0.021), and MAL-QOM (*p* = 0.004). The pairwise test showed pair group differences on those assessments, but the difference between the extended and fast recovery groups on all assessments was not significant.

### 3.3. Results of Logistic Regression Analysis

After screening for association between baseline characteristics and recovery group membership, we used univariate logistic regression analysis to confirm the associations that were significantly related to each characteristic. Table 3 shows the analysis results.

There were no statistically significant predictors for the fast versus extended recovery group membership. For the fast versus limited group membership, all examined predictors, namely, time after stroke, FMA-UE, SIS-Hand, WMFT-Quality, WMFT-Time, MAL-QOM, and MAL-AOU were statistically significant. For the extended versus limited group membership, SIS-Hand, WMFT-Quality WMFT-Time, MAL-AOU, and MAL-QOM were statistically significant.

## 4. Discussion

The Global Burden of Disease Study 2016 estimated the global disability-adjusted life-years due to stroke exceeded 116 million [1]. Multidisciplinary team-based rehabilitation as well as early supported discharge with home-based rehabilitation are effective interventions for increasing survival and functional independency after stroke [2,3,4]. Notably, mirror therapy and robotic therapy and their combinations with unilateral and bilateral conventional therapy were reported as being effective after stroke [16]. In this study, we used FMA-UE scores at pre-intervention, post-intervention, and follow-up to divide participants into groups of UL functional recovery trajectory, as a previous study described [15] and identified predictors associated with the recovery-group membership in our participants with chronic stroke receiving occupational therapy. WMFT, MAL, and SIS-Hand were identified as predictors for the two groups that achieved MCID (fast and extended recovery) versus the group that did not (limited recovery). FMA-UE was also a significant predictor for fast versus limited group membership and approached statistical significance for predicting extended versus limited recovery.

Kline and colleagues (2021) [15] defined their recovery trajectory groups by selecting MCID change (≥10) of the FMA-UE in acute stroke. Their sample distributions in the fast, extended, and limited recovery groups were 19 (47.50%), 12 (30.00%), and 9 (22.50%), respectively. The sample distributions in the current study were 38 (36.19%), 12 (11.43%), and 55 (52.38%), respectively. Limited spontaneous recovery in chronic stroke may explain the higher sample distribution in the limited recovery group of the database that included individuals with chronic stroke. Nevertheless, a significant portion of our participants achieved improvement in FMA-UE after the intervention or at follow-up, demonstrating the recovery potential in the population with chronic stroke.

No differences were found in demographic variables, lesion side, and stroke diagnosis among our recovery groups. Notably, ischemic and hemorrhagic stroke populations were both included in the study, and stroke diagnosis was not significantly different across the three groups of different recovery trajectories. This indicates that the prediction findings were applicable to both stroke types. This was consistent with a study suggesting recovery potential in ischemic and hemorrhagic stroke patients is similar, although the study involved participants with acute stroke [25]. Interestingly, time after stroke was significantly shorter in the fast recovery group compared with the limited recovery group, and time after stroke was found to be a significant predictor of recovery group membership in the logistic regression analysis. This finding was similar to that of previous works that mentioned that the variable of time since onset can be a predictor of an improvement in FMA-UE [11,12,26]. The findings indicated that the principle that faster recovery occurs at a shorter duration after a stroke may also apply to a certain extent to the chronic phase of stroke.

In the univariate logistic regression model, in addition to time after onset, the variables of baseline FMA-UE, WMFT-Quality, WMFT-Time, SIS-Hand, MAL-AOU, and MAL-QOM were significantly associated with recovery group membership. Interestingly, significance powers of the quality scales were higher than those of the time or amount scale; for instance, WMFT-Quality vs WMFT-Time and MAL-QOM vs MAL-AOU. This is consistent with the WMFT-Quality score being more responsive than the WMFT-Time score [21].

Prabhakaran and colleagues found that acute FMA-UE was a predictor of Δ-FMA-UE in 41 stroke survivors by using a linear regression model [7]. Another study used a hierarchical cluster analysis and determined that finger extension, facial palsy, lower-extremity motor function, and Bamford classification can be predictors of Δ-FMA-UE [8]. A study conducted in a stroke population of 93 individuals analyzed NIHSS, FMA-UE, and the Action Research Arm Test as predictors and suggested that FMA-UE was the sole predictor of Δ-FMA-UE. They did not analyze other assessments, such as WFMT and MAL, as predictors [9].

Previously, predictors of MCID changes in FMA-UE were determined in acute [7,9,10], subacute [11], and chronic phases of stroke [5,11,12,13,14]. In the acute phase of stroke, baseline FMA-UE was the best predictor for arm recovery and general disability [10]. In the chronic phase, Semmes–Weinstein Monofilament Examination [5], FMA-UE [5,12,14,26] Hand Movement Scale [11], time since onset [11,12], FIM [12], and the difference in motor threshold between the affected and unaffected hemispheres [13] can be predictors of an improvement in FMA-UE. Notably, Thakkar et al. [12] and Tozlu et al. [13] showed that machine learning methods may enable clinicians to accurately predict an improvement in FMA-UE in the chronic stroke population. Future research may use these methods for outcome prediction studies based on a sufficient sample.

Several lines of evidence have suggested predictors for recovery after stroke rehabilitation [5,7,9,10,11,12,13,14,15]. We are among the first to analyze WMFT and MAL assessments as predictors of fast recovery in motor impairment (i.e., FMA-UE scores) in stroke rehabilitation. The goal of previous research was to predict responders to particular rehabilitations. In this study, we aimed to identify predictors of UL recovery trajectory in patients receiving combinatory interventions based on contemporary approaches to stroke rehabilitation. Our study findings may inform research and practice of contemporary stroke rehabilitation.

There are limitations to this retrospective study. Potentially relevant predictors (e.g., self-efficacy and proxy support) warrant future scrutiny. Second, our study findings need to be validated in an independent, prospective cohort. In addition, the study sample in the extended recovery group is limited. This may partially explain why there are no significant predictors for fast versus extended group membership in this study. Further investigations are needed to validate the present findings based on a larger sample.

## 5. Conclusions

In order to understand predictors for UL functional recovery trajectory in chronic stroke, we applied the recovery trajectory model defined by Kline et al. [15] for acute stroke and identified factors associated with group membership in this population. We found that WMFT, MAL, SIS-Hand, and FMA-UE were predictors for the two groups that achieved MCID after intervention or at follow-up (fast and extended recovery) versus the group that did not (limited recovery). These findings may assist healthcare professionals in making optimal therapeutic decisions and in accurately informing clients and caregivers about the UL functional recovery after stroke rehabilitation.

## Figures and Tables

**Table 1:** Demographic and stroke-related characteristics.

Characteristics	Mean ± SD, n (%), or Median (Q1–Q3)
Age at time of stroke (years)	55.68 ± 11.42
Male sex	70 (66.67)
Years of education	12 (9–14)
Side of hemiplegia (right)	61 (58.10)
Type of stroke (hemorrhagic)	47 (44.76)
Time after stroke onset (month)	14 (7–31)
NIHSS score	4 (3–6)

Abbreviation: NIHSS, National Institutes of Health Stroke Scale; Q1, quartile 1; Q3, quartile 3; SD, standard deviation.

**Table 2:** Descriptive and inferential statistics of baseline measurements among recovery groups.

	Limited Recovery (n = 55)	Extended Recovery (n = 12)	Fast Recovery (n = 38)	*p* Value
Age at stroke (years)	54.11 ± 10.97	55.81 ± 11.98	57.90 ± 11.83	0.29
Male sex	39 (70.91)	7 (58.33)	24 (63.16)	0.60
Educated years	12.00 (9.00–15.75)	12.00 (8.25–14.25)	12.00 (9.00–14.00)	0.37
Side of hemiplegia (right)	32 (58.18)	7 (58.33)	22 (57.89)	0.99
Stroke diagnosis (hemorrhagic)	28 (50.91)	2 (16.67)	17 (44.74)	0.09
Time after stroke (months) ^a^	20.00 (10.00–41.00)	11.00 (3.75–44.00)	11.50 (6.00–18.00) ^c^	0.005 *
NIHSS	4.00 (3.00–6.75)	3.50 (2.00–4.50)	4.00 (3.00–6.00)	0.45
FMA-UE ^a^	27.50 (25.00–31.75)	34.50 (28.25–38.25) ^b^	34.00 (27.00–42.00) ^c^	0.007 *
MAS	0.89 (0.63–1.14)	0.91 (0.62–1.01)	0.82 (0.50–1.04)	0.62
WMFT (quality) ^a^	2.13 (1.88–2.38)	2.70 (2.22–3.00) ^b^	2.80 (2.07–3.07) ^c^	<0.001 *
WMFT (time) ^a^	14.22 (10.31–18.01)	11.35 (6.66–13.42) ^b^	10.05 (5.13–15.14) ^c^	0.002 *
SIS-Hand ^a^	15.00 (5.00–35.00)	35.00 (7.50–51.25)	35.00 (15.00–55.00) ^c^	0.007 *
MAL-AOU ^a^	0.80 (0.53–1.04)	1.40 (0.70–2.36)	1.11 (0.72–1.83) ^c^	0.021 *
MAL-QOM ^a^	0.48 (0.25–0.77)	1.24 (5.00–2.24) ^b^	0.81 (0.48–1.73) ^c^	0.004 *
NEADL	28.00 (15.25–43.75)	35.00 (24.25–45.75)	28.00 (18.00–44.00)	0.69

Data are presented as n (%), mean ± standard deviation, or as median (quartile 1-quartile 3). * *p* < 0.05 between recovery groups by Kruskal–Wallis test. ^a^ *p* < 0.05 between groups by using logistic regression. ^b^ *p* < 0.05 between limited and extended recovery groups by Wilcoxon rank sum tests. ^c^ *p* < 0.05 between limited and fast recovery groups by Wilcoxon rank sum tests. Abbreviations: AOU, Amount of use; FMA-UE, Fugl–Meyer Assessment-Upper Extremity subscale; MAL, Motor Activity Log; MAS, Motor Assessment Scale; NEADL, Nottingham Extended Activities of Daily Living; NIHSS, National Institutes of Health Stroke Scale; QOM, Quality of Movement; SIS, Stroke Impact Scale; WMFT, Wolf Motor Function Test.

**Table 3:** Results of logistic regression models.

Baseline Characteristics	β	*p* Value	Odds Ratio (95% CI)
Fast vs extended recovery			
Time after stroke onset	−0.01	0.955	1.02 (0.96–1.02)
FMA-UE	0.01	0.806	1.01 (0.93–1.09)
WMFT-Quality	−0.09	0.883	0.91 (0.28–3.03)
WMFT-Time	0.03	0.689	1.03 (0.90–1.17)
SIS-Hand	0.001	0.961	1.00 (0.98–1.03)
MAL-AOU	−0.28	0.390	0.75 (0.39–1.44)
MAL-QOM	−0.34	0.344	0.72 (0.36–1.43)
Fast vs limited recovery			
Time after stroke onset	−0.03	0.024	0.97 (0.95–1.00)
FMA-UE	0.08	0.003	1.09 (1.03–1.15)
WMFT-Quality	1.68	<0.001	5.37 (2.17–13.33)
WMFT-Time	−0.11	0.007	0.90 (0.83–0.97)
SIS-Hand	0.03	0.002	1.03 (1.01–1.05)
MAL-AOU	0.62	0.027	1.86 (1.07–3.24)
MAL-QOM	0.84	0.006	2.32 (1.28–4.21)
Extended vs. limited recovery			
Time after stroke onset	−0.01	0.37	0.99 (0.96–1.02)
FMA-UE	0.08	0.065	1.08 (1.00–1.12)
WMFT-Quality	1.77	0.006	5.88 (1.67–20.73)
WMFT-Time	−0.13	0.035	0.88 (0.77–0.99)
SIS-Hand	0.03	0.034	1.03 (1.00–1.06)
MAL-AOU	0.91	0.013	2.48 (1.21–5.05)
MAL-QOM	1.18	0.003	3.24 (1.49–7.03)

Abbreviations: AOU, Amount of use; CI, confidence interval; FMA-UE, Fugl–Meyer Assessment-Upper Extremity subscale; MAL, Motor Activity Log; QOM, Quality of Movement; SIS, Stroke Impact Scale; WMFT, Wolf Motor Function Test.

## Data Availability

The data presented in this study are available on request from the corresponding author. The data are not publicly available because, according to the Personal Information Protection Act enacted in Taiwan, individualized data cannot be released for the protection of privacy.
